# Properties of sodium currents in neonatal and young adult mouse superficial dorsal horn neurons

**DOI:** 10.1186/s12990-015-0014-5

**Published:** 2015-03-28

**Authors:** Melissa A Tadros, Kristen E Farrell, Brett A Graham, Alan M Brichta, Robert J Callister

**Affiliations:** School of Biomedical Sciences and Pharmacy, Faculty of Health and Medicine and Hunter Medical Research Institute, The University of Newcastle, Callaghan, Newcastle, NSW 2308 Australia

**Keywords:** Development, Activation, Spinal cord, Pain, Action potential

## Abstract

**Background:**

Superficial dorsal horn (SDH) neurons process nociceptive information and their excitability is partly determined by the properties of voltage-gated sodium channels. Recently, we showed the excitability and action potential properties of mouse SDH neurons change markedly during early postnatal development. Here we compare sodium currents generated in neonate (P0-5) and young adult (≥P21) SDH neurons.

**Results:**

Whole cell recordings were obtained from lumbar SDH neurons in transverse spinal cord slices (CsF internal, 32°C). Fast activating and inactivating TTX-sensitive inward currents were evoked by depolarization from a holding potential of −100 mV. Poorly clamped currents, based on a deflection in the IV relationship at potentials between −60 and −50 mV, were not accepted for analysis. Current density and decay time increased significantly between the first and third weeks of postnatal development, whereas time to peak was similar at both ages. This was accompanied by more subtle changes in activation range and steady state inactivation. Recovery from inactivation was slower and TTX-sensitivity was reduced in young adult neurons.

**Conclusions:**

Our study suggests sodium channel expression changes markedly during early postnatal development in mouse SDH neurons. The methods employed in this study can now be applied to future investigations of spinal cord sodium channel plasticity in murine pain models.

## Background

Superficial dorsal horn (SDH; laminae I-II) neurons are important for spinal processing of sensory information, including thermal, pruritic, light touch, and nociceptive inputs. The excitability of this population is determined by a variety of voltage-activated conductances including voltage-gated sodium channels, which play a critical role in determining action potential (AP) discharge. We have previously shown that several AP properties change during early postnatal development in mice [1]. Most notably, AP amplitude and the pattern of AP discharge change markedly between the first and third weeks of postnatal development in SDH neurons, implying that sodium current properties may be altered over this period.

To date, nine sodium channel subtypes have been described [2] and four of these (Na_v_1.1, 1.2, 1.3 and 1.6) are present in the rodent SDH [3,4]. Several studies have assessed mRNA expression for these subunits during early postnatal development. Collectively, they have shown an increase in Na_v_1.1 and a concurrent decrease in Na_v_1.2 and 1.3 during the first three postnatal weeks, with little or no change in the expression of Na_v_1.6 [5-7]. Thus, molecular evidence suggests sodium channel expression, as assessed by mRNA levels, changes during the first three postnatal weeks of development.

In contrast to our molecular understanding, only limited data exists on the electrophysiological properties of sodium channels in rodent SDH neurons during development. Undertaking voltage clamp analysis of sodium channel properties in SDH neurons is difficult because of the rapid kinetics of these channels and the complex dendritic structure of SDH neurons [8,9]. These problems, which make achieving adequate voltage clamp difficult, have been partially overcome by studying sodium channel properties in dorsal horn neurons (laminae I-III) using an extracted soma method [10]. Studies applying this approach in the rat have shown that sodium current amplitude is small and constant in the soma during the first six weeks of postnatal development. In contrast, sodium currents recorded in intact neurons show a more than two-fold increase in amplitude over this period [11]. Recently, the presence of persistent sodium currents has been demonstrated in “pacemaker neurons” of the newborn rat SDH [12]. Here spontaneous AP discharge, associated with a persistent sodium current, is thought to be crucial for shaping circuit formation as occurs in other CNS pathways such as those of the visual system [13]. Despite these data from rat, which highlight the importance of sodium currents during development, similar detailed information for sodium currents in intact SDH neurons are lacking for the mouse.

Here we examine the properties of sodium currents in intact SDH neurons either side of a critical period in the development of neuron excitability and AP discharge in the mouse. There were two principle motivations for this study: the increasing recognition of the need to study native channels expressed in intact neurons [14,15], and the increasing use of mutant and transgenic mice in studies to examine pain-processing mechanisms in the SDH [16-19]. We use a method described by Magistretti et al. [20] to restrict our analysis to SDH neurons where adequate voltage clamp was achieved. This technique involves generating current voltage (I/V) curves for each neuron and eliminating those with poorly clamped currents. Based on sodium currents recorded in intact SDH neurons in neonatal (P0-P5) and young adult (≥ P21) mice, where adequate space clamp was achieved, we show that sodium current expression increases more than two fold between the first and third postnatal weeks. This is also accompanied by other changes in current properties including slower recovery from inactivation and decreased tetrodotoxin (TTX) sensitivity in neurons from young adults compared to neonates.

## Results

**Figure 1:**
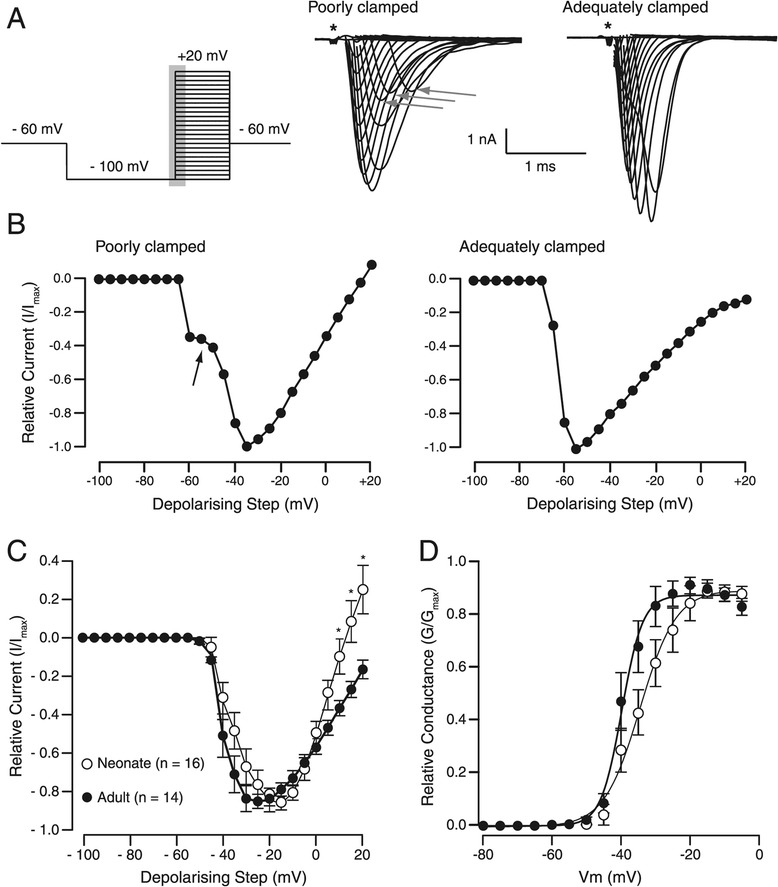
**Selection of cells for detailed analysis. A**. Left panel shows the activation protocol, applied in voltage clamp from a holding potential of −60 mV. A pre-pulse to −100 mV (100 ms duration) was followed by a series of depolarising steps (5 mV increments, 50 ms duration, −100 to +20 mV). Grey shaded area indicates the time period (~5 s) over which the right traces were captured for analysis. Centre and left panels show representative traces from poorly- and adequately clamped adult SDH neurons. Capacitive currents have been removed for clarity (asterisk). **B**. Current–voltage (I-V) plots for the recordings shown in A. Arrow in left plot indicates the deflection that characterizes poorly clamped currents (eg, centre panel of A, arrows). **C**. Group data showing I-V plots generated from adequately clamped currents in neonatal (open circles) and adult SDH neurons (filled circles). Data for each cell have been normalized to peak current and plotted against membrane potential. Sodium currents in neonates function over a narrower membrane potential range than those from adults. **D**. Relative conductance plotted as a function of voltage for neonates (open circles) and adult SDH neurons (filled circles).

A total of 215 recordings were obtained; 84 from neonatal (P0-5) and 131 from young adult (≥ P21) SDH neurons. Most recordings, in both age groups, (81% for neonates and 90% for young adults) showed signs of poor space clamp (Figure 1). Such neurons were excluded from further analysis, leaving 16 neonatal and 14 young adult “adequately clamped” SDH neurons for analysis. Poor space clamp was determined by careful inspection of the currents collected at potentials between −60 and −40 mV. Poorly clamped neurons showed little increase in inward current amplitude during successive depolarising steps (Figure 1A; middle panel) and often showed a pronounced delay between membrane depolarisation and current activation. After I-V curves were generated for each neuron, these poorly clamped currents resulted in a marked deflection in the IV relationship at potentials between −60 and −50 mV (Figure 1B) [20]. Application of TTX (1 μM for 2 min; not shown) completely abolished the currents produced in response to the activation protocol (n = 5 for neonates and young adults) thereby confirming that the inward currents we observed were generated by TTX-sensitive sodium channel activity.

### Activation of sodium currents

I-V curves (Figure 1C) were generated from neurons with adequate space clamp (n = 16 and 14 in neonates and young adults, respectively) using the data obtained from the activation protocol shown in Figure 1A. In neonates, the activation potential and maximal current occurred at slightly more depolarised potentials compared to young adults. The slope of the I-V curve after peak current was reached decreased in young adults. Conductance was calculated for voltage commands from −80 to 0 mV (Figure 1D). Although the curve was shifted to the right in neonates, half maximal conductance (G/G_Max_; −33.83 ± 2.24 vs. -37.34 ± 1.88; neonates vs. adults) and the slope (k; 2.03 ± 0.36 vs. 1.91 ± 0.64) did not differ. These data suggest subtle changes in sodium current activation in SDH neurons from young adults vs. neonates.

### Sodium current properties

**Figure 2:**
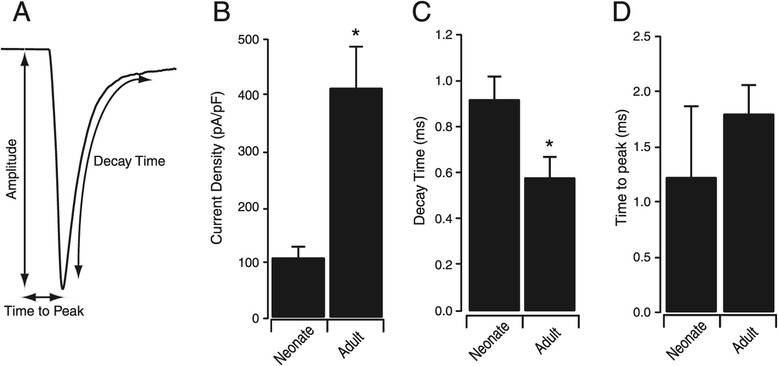
**Sodium current properties in neonatal and adult SDH neurons. A**. Representative sodium current illustrating origin of measurements shown in bar plots. All measurements were made on the largest current observed in response to the activation protocol shown in Figure 1A. **B-D**. Bar plots comparing current density **(B)**, decay time **(C)** and time to peak **(D)** for neonates and adults. Current density was greater and decay time was faster in adults (* p < 0.05). Time to peak was similar for both age groups.

Peak amplitude, time to peak and the decay time were measured on the largest current obtained for each neuron during the activation protocol (Figure 2A). Peak amplitude was greater in young adults (2.03 ± 0.62 nA vs. 5.21 ± 0.83 nA, neonates vs. young adults, p = 0.004). Although current density was significantly larger in adults (107.3 ± 19.8 pA/pF vs. 412 ± 74.3 pA/pF, p < 0.01, Figure 2B), this was not accompanied by a change in capacitance between neonates and adults (17.0 ± 1.65 pF vs. 15.0 ± 1.76 pF), suggesting this difference was due to a change in the levels of channel expression. The decay time constant, measured from 90% to 10% of the peak amplitude, was slower in neonates (0.92 ± 0.1 ms vs. 0.58 ± 0.09 ms, p = 0.02, Figure 2C). Time to peak did not differ between neonates and adults (1.22 ± 0.64 ms vs. 1.80 ± 0.26 ms, p = 0.07, Figure 2D). Thus, sodium currents display an increase in amplitude and faster inactivation kinetics between the first and third postnatal weeks.

### Steady state inactivation of sodium currents

**Figure 3:**
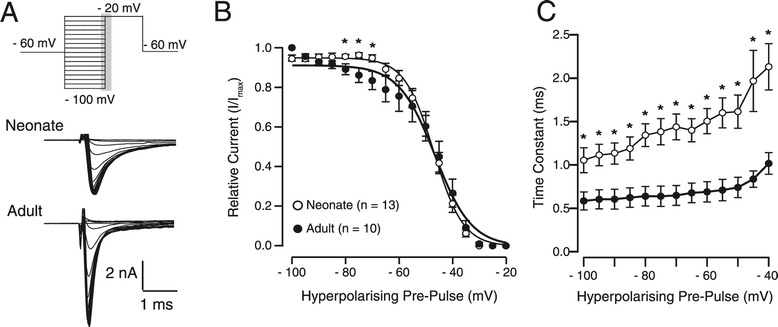
**Steady state inactivation of sodium currents in neonates and adults. A**. Top trace shows the inactivation protocol, applied in voltage clamp from a holding potential of −60 mV. A series of hyperpolarising pre-pulses (5 mV increments, 100 ms duration, −100 to −20 mV) were followed by a depolarising step (to −20 mV, 50 ms duration). Lower traces show representative responses from the time period shown in grey (upper trace) are illustrated for neonates and adults. Capacitive currents have been removed. **B**. Inactivation curves for neonatal (open circles) and adult SDH neurons (filled circles). Steady state inactivation is similar in both groups, however, neonatal currents do not begin to inactivate until slightly more depolarized membrane potentials (* p < 0.05). **C**. Time constants of inactivation plotted as a function of voltage for neonates (open circles) and adults (filled circles). Neonates displayed greater time constants of inactivation for all voltages between −100 and −40 mV.

Steady state inactivation curves were generated from data collected in response to the inactivation protocol shown in Figure 3A. Although these curves show reduced steady state inactivation in neonatal SDH neurons at membrane potentials between −80 to −60 mV (Figure 3B), inactivation was remarkably similar between the two groups for the remaining range of voltages examined (−60 and −20 mV). Furthermore, Boltzmann curve fits to these data revealed no significant differences in half inactivation (V_H_; −47.40 ± 1.35 mV vs. −46.94 ± 2.94 mV; neonates vs. young adults) or the rate of inactivation (k; −5.08 ± 0.41 pA/mV vs. −4.99 ± 0.49 pA/mV). This suggests steady state inactivation is similar in neonates and young adults except at more hyperpolarized membrane potentials where it is more pronounced in young adults (−80 to −60 mV). Time constants of inactivation were measured at voltage commands ranging from −100 to −40 mV, with neonates displaying larger time constants at all voltages compared to adults (p < 0.01, Figure 3C). There was also a greater deviation in time constant between −100 and −40 mV for neonates compared to adults. Overall, these data suggest that whilst subtle changes occur in steady state inactivation, the inactivation time constants decrease drastically from the first to third postnatal week.

### Recovery from inactivation

**Figure 4:**
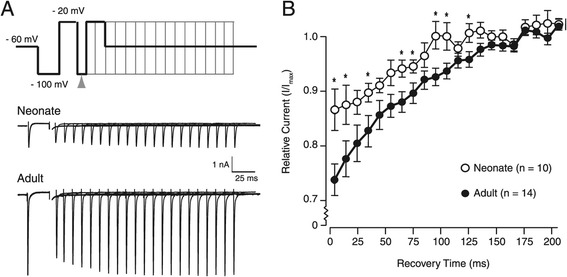
**Recovery from inactivation in neonates and adults. A**. Upper trace shows the two-pulse protocol applied in voltage clamp from a holding potential of −60 mV. A hyperpolarising pre-pulse (−100 mV, 100 ms duration) was followed by a depolarising step (−20 mV, 50 ms duration). Next a hyperpolarising ‘recovery’ step of increasing duration (−100 mV, 5 to 200 ms, 5 ms increments, indicated by arrowhead) was applied before a final second depolarising step (−20 mV). Lower two traces show responses recorded for neonatal and adult SDH neurons. Capacitive currents not show. **B**. Plots of current (normalized to peak current) versus recovery time for neonatal (open circles) and adult recordings (filled circles). Note the faster recovery from inactivation in neonates (* p < 0.05).

Recovery from inactivation was measured using a two-pulse protocol where the recovery time between pulses was incrementally increased (Figure 4A). Recovery from inactivation was faster in neonates, with ~ 85% recovery observed after the first 5 ms in neonates compared to ~ 75% in young adults (p < 0.01). The time taken for full recovery from inactivation was also less in neonates (~100 ms vs. ~ 150 ms; Figure 4B).

### TTX sensitivity of sodium currents

**Figure 5:**
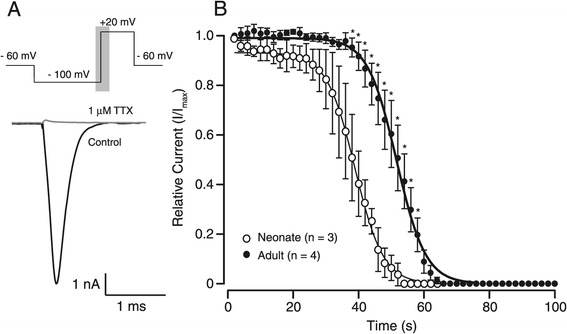
**TTX sensitivity of sodium currents from neonatal and adult SDH neurons. A**. Upper trace shows protocol (repeated 0.5 Hz) applied to neonatal and adult SDH neurons during bath application of 1 μM TTX. Bottom trace shows representative maximum sodium current recorded in an adult SDH neuron (black trace) before and after application of 1 μM TTX for 2 mins (grey trace). Both traces were recorded during the onset of the depolarising step (grey shading in top panel). **B**. Plots of peak normalized current versus time during application of 1 μM TTX. Sigmoid curves show the onset of TTX block was more rapid in neonates (* p < 0.05), however, the slope of the relationship was similar in neonatal and adult recordings.

Sodium currents, measured in expression systems and dorsal root ganglia (DRG), can be divided into TTX sensitive (TTX-S) and TTX resistant (TTX-R) according to their response to tetrodotoxin (TTX) [21]. Moreover, these channel types are developmentally regulated and can change following nerve injury in DRG neurons [22,23]. We therefore compared the TTX sensitivity of sodium currents in a subset of neurons from neonates and young adults (Figure 5). TTX (1 μM) was applied to examine how rapidly peak sodium current amplitude decreased. Sigmoid curves fitted to the TTX sensitivity data (Figure 5B) revealed a rightward shift in the young adult TTX sensitivity plot. Specifically, the time to half peak current was shifted to the right in young adults (I/I_max_; 38.25 ± 2.90 s vs. 51.37 ± 2.13 s, neonatal vs. young adult, p = 0.01). The slope of the relationship did not change (−4.14 ± 0.44 pA/s vs. –4.06 ± 0.60 pA/s). These data suggest sodium currents in neonatal SDH neurons are more sensitive to TTX and/or they express a higher proportion of TTX-S sodium channels. The available literature, however, suggest all major sodium channel types found in the SDH are TTX-S [24,25].

## Discussion

This study was prompted by our previous work describing developmental changes in the excitability of mouse lumbar SDH neurons [26]. One possible contributor to these changes is altered expression and properties of voltage gated sodium channels [24,27]. Here, we report differences in sodium current properties between neonatal and young adult lumbar SDH neurons. Importantly, we made whole cell patch clamp recordings in intact SDH neurons and only studied those neurons where good space clamp was achieved. Our main finding is that there is a greater than two-fold increase in sodium channel expression between the first and third postnatal weeks. This is accompanied by subtle changes in activation range, steady-state inactivation, recovery from inactivation, and TTX sensitivity. Below we discuss the caveats associated with our experiments, our main finding, and the factors that might explain some of the changes in sodium channel properties that occur in early postnatal development.

In the majority of our sodium current recordings we were unable to achieve adequate voltage clamp, presumably due to the dendritic complexity and heterogeneity in SDH neurons. Similar space clamp issues have been reported for fast currents in other intact neurons [20,28-30]. We carefully selected neurons where adequate space clamp was achieved using the method proposed by Magistretti et al. [20]. This resulted in a low yield of recordings for analysis (Figure 1). Another technical consideration is the increasing size of the dendritic trees of SDH neurons associated with postnatal development [31]. This factor would result in more rejected neurons in the older age group because of space clamp issues. Indeed, the incidence of “rejected” neurons was slightly greater in (90% vs. 81%) in young adult neurons. Thus, some of our comparisons between neonatal and young adult SDH neurons may be influenced by age-related sampling bias. Differences in TTX-sensitivity between neonatal and adult SDH neurons were also observed (Figure 5). Whilst this might be a developmental difference, there is also the possibility that TTX access was decreased in adult slices because of the increased levels of myelination in the older spinal cords [32].

Our major finding is that peak sodium current more than doubles in mouse SDH neurons over the first 3–4 weeks of postnatal development. Previous work on intact rat lamina I-III neurons has also shown that sodium current peak amplitude increases over the first six weeks of postnatal development (P0 - P39; 11). In the rat study the authors measured peak current amplitude at six time points and showed current amplitude increased linearly at a rate of 83 pA/day. Assuming a linear rate of channel insertion in the mouse, a similar calculation using mean ages for our P0-5 and ≥ P21 groups results in a rate of ~150 pA/day for mouse SDH neurons. This is almost double the rate in rats and suggests sodium channel insertion proceeds more rapidly in the mouse after birth. It must be noted however that voltage escape, which would reduce peak current amplitude, was not controlled in the rat sample and the experiments were undertaken at lower temperatures (20-24°C vs. 32°C). Nevertheless, this comparison between the two rodent species suggests mouse SDH neurons proceed towards “electrical maturity” faster than those in the rat.

In addition to changes in peak amplitude, we also noted a decrease in current decay time with age (Figure 2C and 3C). This would shorten repriming times for young adult sodium channels and allow higher AP discharge frequencies. To our knowledge the only data available on SDH neuron AP discharge frequency during development are for tonic firing neurons in rat. In this species, firing frequency does not change between P3 and P21 [33], suggesting sodium channel kinetics does not affect AP discharge frequency in tonic firing neurons at least. This may be a species-specific finding, as the proportion of the various cells types (ie, SDH heterogeneity) changes significantly during SDH development in mice [1]. Thus, further investigation into the relationship between sodium current properties and AP discharge frequencies in the mouse is required.

We also observed subtle differences in activation, steady state inactivation, and recovery from inactivation in neonate and adult sodium channels. To our knowledge these properties have not be studied in intact dorsal horn neurons. Sodium channels in the mammalian CNS consist of nine pore forming α and four auxiliary β subunits [2,34]. Their expression and combination can shape channel properties and both α and β subunits are developmentally regulated [35]. Molecular analyses of sodium channel expression in the rat spinal cord indicate that subunit expression changes in early postnatal development: most notably, Na_v_1.1 levels increase and Na_v_1.2 and Na_v_1.3 decrease [4-6]. Na_v_1.3 subunit levels change most dramatically during development. This subunit is highly expressed in the neonatal rat dorsal horn and shows a 3.9-fold decrease between P3 and P49 [7]. The Na_v_1.3 subunit facilitates recovery from inactivation and has been proposed to underlie the hyperexcitability observed in DRG neurons after injury [22,36]. Thus, the faster recovery from inactivation we observed in mouse neonatal SDH neurons is consistent with the developmental expression profile and known function of Na_v_1.3 channels (Figure 4B).

In addition to the enhanced sodium current amplitude, there was a modest shift to the left for the conductance (Figure 1D) and steady state inactivation increased at membrane potentials between −80 to −60 mV (Figure 3B) during postnatal development. These data cannot be simply explained by changes in subunit composition during development as all subunits present in the mammalian dorsal horn (ie, Na_v_1.1, Na_v_1.2, Na_v_1.3 and Na_v_1.6) exhibit remarkably similar activation and steady state inactivation, at least for human α subunits expressed in HEK cells [37]. Developmentally regulated splicing is also known to occur in some α subunits, resulting in channels with altered kinetics. For example, Na_v_1.2 isoforms (II and IIA) isolated from rat brain, show a depolarising shift in the I-V relationship when expressed in Xenopus oocytes [38]. Interestingly, the expression of these Na_v_1.2 isoforms (II and IIA) as well as the Na_v_1.6 subunit are developmentally regulated during the first postnatal month in the rat brain and spinal cord [7,39]. Post-translational modifications via phosphorylation can also change Na_v_1.6 channels properties [40]. Thus, alternative splicing or post-translational modifications of subunit proteins may explain some of the subtle changes we observed in sodium channel properties.

The changes we observed in some sodium channel properties could also be due to the considerable electrophysiological heterogeneity that exists in both neonatal and adult SDH neurons in rodents [26,41,42]. SDH neurons have been grouped into at least four types according to their AP discharge profile in response to square step current injection and sodium currents play an important role in determining both AP and discharge properties. For example, tonically discharging neonatal and adult mouse SDH neurons have lower rheobase currents, larger AP amplitudes and more hyperpolarized AP thresholds than those that only discharge a single spike [1,26,43,44]. Furthermore, in adult rat SDH adapting-firing neurons display smaller sodium currents than tonic firing neurons [45]. Unfortunately, the recording conditions we used precluded assessment of firing categories and it is likely that some of the subtle changes we found are a result of “averaging” across SDH neuron subtypes [41]. This issue could be directly addressed in future studies by preferentially targeting neurons known to have certain AP discharge patterns. For example, parvalbumin-positive SDH neurons are predominately tonic firers or initial bursters [8] and projection neurons identified by back-labelling from brainstem nuclei exhibit gap and burst firing [46].

## Conclusions

Given the central role of sodium currents for AP generation and signal propagation, along the ascending pain pathway, the expression and characteristics of sodium channels has been investigated in a variety of pain models. This is especially true for sensory neurons (DRGs) in both animals and humans where injury or inflammation leads to hyperexcitability [47,48]. For DRGs it is clear that nerve injury increases sodium channel density and alters subunit expression and properties [47]. This upregulation facilitates rapid recovery from inactivation of TTX-S currents in DRG neurons [14]. In the adult rat spinal cord, axotomy, spinal contusion, and peripheral nerve injury all result in upregulation of Na_v_1.3 [36,49]. These observations have lead to suggestions that pharmacological targeting of specific sodium channel isoforms in DRG neurons could be used to treat chronic pain. Our work now provides a baseline dataset for similar studies to be undertaken in SDH neurons, examining the potential contribution of sodium channels to the increased excitability and central sensitization in models of pathological pain.

## Methods

### Preparation of spinal cord slices

The University of Newcastle’s Animal Care and Ethics Committee approved all procedures. Mice (C57Bl/6; both sexes) were divided into two age groups: P0-5 and ≥ P21 (hereafter termed neonates and young adults, respectively). These age ranges were chosen because they are either side of a previously described critical period in the development of electrophysiological properties in SDH neurons [1,26]. Approximately equal numbers of males and females were used (55% vs. 45%, respectively).

Neonatal mice were immersed in ice to induce hypothermic anaesthesia and young adult mice were anaesthetised with Ketamine (100 mg/kg i.p.). Animals from both groups were decapitated and the vertebral column rapidly isolated and immersed in ice-cold oxygenated sucrose substituted artificial cerebrospinal fluid (sACSF) containing (in mM): 250 sucrose, 25 NaHCO_2_, 10 glucose, 2.5 KCl, 1 NaH_2_PO_4_, 1 MgCl_2_ and 2.5 CaCl_2_. The sACSF was continually bubbled with 95% O_2_ – 5% CO_2_ to maintain a pH of 7.3 – 7.4. The lumbosacral enlargement was removed and positioned on a Styrofoam support block (rostral side down). The enlargement and block were secured to a cutting stage with cyanoacrylate glue (Loctite 454, Loctite, Caringbah, NSW, Australia). Transverse spinal cord slices (300–400 μm thick) were obtained using a vibratome (VT1200S, Leica Microsystems, Wetzlar, Germany). Slices were transferred to an interface storage chamber containing oxygenated ACSF (118 mM NaCl substituted for sucrose in sACSF) and allowed to recover for 1 hour at room temperature (22 – 24°C) before recording commenced.

### Electrophysiology

Individual slices were transferred to a recording chamber (volume 0.4 mls) and continually superfused (4–6 bath volumes/min) with oxygenated ACSF. The recording bath was maintained at near-physiological temperature (32°C) using an in-line heating device (Model TC-324B, Warner Instruments, Hamden, CT). Using a Multiclamp 700B Amplifier (Molecular Devices, Sunnydale, CA), whole cell patch clamp recordings were obtained from SDH neurons, visualised using infrared differential contrast optics and an IR sensitive camera (Rolera-XR, Olympus, NJ). In order to record sodium currents in isolation, outward potassium currents were blocked by using a caesium fluoride based internal containing (in mM): 140 CsF, 1 EGTA, 10 NaCl and 10 HEPES, adjusted to pH 7.3 with CsOH [28].

Once the whole cell recording configuration was established, whole cell capacitive currents were balanced and series resistance was compensated by 60–70%. Membrane potential was held at −60 mV and a series of protocols were applied in voltage clamp. Activation of sodium currents was assessed using an initial hyperpolarising pre-pulse (to −100 mV, 100 ms duration) to deactivate voltage sensitive sodium channels, immediately followed by a series of depolarising voltage steps (−100 mV to +20 mV, 5 mV increments, 50 ms duration). Sodium current inactivation was assessed using a series of deactivating hyperpolarising pre-pulses (−100 mV to +20 mV, 5 mV increments, 100 ms duration), immediately followed by a depolarising voltage step (to −20 mV, 50 ms duration). Finally, a two-pulse protocol was applied to assess recovery from inactivation. This consisted of an initial hyperpolarising pre-pulse (to −100 mV, 100 ms duration) and then a depolarising voltage step (to −20 mV, 50 ms duration). This was immediately followed by a second ‘recovery’ hyperpolarising step (to −100 mV) of increasing duration (5 to 200 ms, 5 ms increments), before a second depolarising voltage step (to −20 mV, 50 ms duration).

In some neurons, tetrodotoxin (TTX; Alomone Laboratories, Jerusalem, Israel) sensitivity was assessed by measuring the peak sodium current every two seconds during bath application of 1 μM TTX. As above, sodium currents were activated using a hyperpolarising pre-pulse to −100 mV, 100 ms duration, followed by a depolarising voltage step to −20 mV for 50 ms.

### Data capture and analysis

Data were digitised online (filtered at 6 kHz, sampled at 50 kHz) via an ITC-16 A/D board (Instrutech, Long Island, NY) and stored on a Macintosh computer using Axograph X software (Axograph X, Sydney, Australia). All data were analysed offline using the Axograph X software.

Current amplitude was measured at the maximum negative peak of each trace and current density was calculated by dividing amplitude by the neuron’s capacitance. Time to peak was measured on the largest current generated during the activation protocol. Current decay time constants were obtained from single exponential fits over 90% to 10% of the inactivation phase of the current (see Figure 2A). Data were normalised to the maximum current observed in each protocol. Current–voltage (I-V) curves were generated by plotting normalised peak current against membrane voltage. Conductance was calculated for voltage commands from −80 to 0 mV, using the equation G = I/(V_command_-E_Na_) with E_Na_ set to +70 mV (32°C, Nernst Equation). In order to assess recovery from inactivation, the peak amplitude of the second inward current was compared to the first. TTX sensitivity was assessed by plotting peak current amplitude against time. Boltzmann curves were fit to conductance and steady state inactivation plots, using the equation g/g_max_ = 1/[1 + exp(V-V_H_)/k] where g/g_max_ is normalised conductance, V is membrane voltage, V_H_ is voltage at half maximal conductance or inactivation and k is the slope factor.

SPSS v18 software package (SPSS, Chicago, IL) was used for statistical analysis. Student’s T-tests were used to compare neonate and young adult data. Statistical significance was set at p < 0.05 and all data are presented as means ± SEM.

## Abbreviations

SDH: Superficial dorsal horn
AP: Action potential
ACSF: Artificial cerebrospinal fluid
TTX: Tetrodotoxin
TTX-S: Tetrodotoxin-sensitive
TTX-R: Tetrodotoxin-resistant
DRG: Dorsal root ganglia
